# Effect of Dexamethasone Combination with Gentamicin in Chemical Labyrinthectomy on Hearing Preservation and Vertigo Control in Patients with Unilateral Meniere’s Disease: A Randomized Controlled Clinical Trial

**DOI:** 10.3390/jcm10235581

**Published:** 2021-11-27

**Authors:** Seong-Hoon Bae, Jeon-Mi Lee, Hyun-Jin Lee, Gina Na, Sung-Huhn Kim

**Affiliations:** 1 Department of Otorhinolaryngology, Yonsei University College of Medicine, Seoul 03722, Korea; bshsap1@yuhs.ac; 2 Department of Otorhinolaryngology, Ilsan Paik Hospital, Inje University College of Medicine, Goyang 10380, Korea; entmeowmiya@gmail.com (J.-M.L.); kalosophiana@gmail.com (G.N.); 3 Department of Otorhinolaryngology–Head and Neck Surgery, Incheon St. Mary’s Hospital, College of Medicine, The Catholic University of Korea, Seoul 21431, Korea; idgenesis@naver.com

**Keywords:** Meniere’s disease, chemical labyrinthectomy, dexamethasone, gentamicin

## Abstract

Chemical labyrinthectomy using gentamicin is a popular method for treating intractable vertigo attacks in Meniere’s disease. However, the risk of hearing loss remains a major concern for clinicians. We investigated the effect of simultaneous dexamethasone and gentamicin application on hearing preservation and vertigo control in patients with intractable unilateral Meniere’s disease. A single-institutional, prospective, single-blinded, randomized clinical trial was conducted. Gentamicin-soaked Gelfoam^®^ was directly applied on the oval window following middle ear exploration. On the round window, dexamethasone-soaked Gelfoam^®^ was applied in the gentamicin with dexamethasone group (GD group, *n* = 18), and saline-soaked Gelfoam^®^ was applied in the gentamicin with sham reagent group (GO group, *n* = 19). The hearing change 8 weeks after the procedure and vertigo control 2–12 months after the procedure were investigated. The high-frequency hearing threshold was significantly increased in the GO group (*p* = 0.005 and 0.012 for 4 and 8 kHz, respectively), but not in the GD group. The short-term (2–6 months) vertigo control was more successful in the GD group (57.89% vs. 94.44%, *p* = 0.019), but long-term control (6–12 months) was insignificant. In conclusion, the combined application of gentamicin and dexamethasone in chemical labyrinthectomy is an effective method for protecting high-frequency hearing and vertigo control.

## 1. Introduction

Meniere’s disease (MD) is an inner ear disorder characterized by episodic vertigo, fluctuating sensorineural hearing loss combined with aural fullness, and/or tinnitus [1]. The most annoying and concerning symptom of this disease is unpredictable, acute, and recurrent vertigo attacks. Therefore, most of the medical and surgical treatments are focused on controlling vertigo attacks rather than hearing symptoms [2,3]. The first-line treatment is medication and lifestyle modification. Although there is insufficient scientific evidence to support this first-line treatment, patients are generally prescribed medications such as betahistidine and/or diuretics and recommendations for lifestyle modification, such as a low-salt diet and avoidance of excessive stress, alcohol, smoking, and caffeine. The most commonly used second-line treatment for non-responders is intratympanic corticosteroid (ITC) injection. However, patients who suffer from intractable vertigo even after second-line treatment require tertiary treatment [2]. There are several invasive treatment options for these patients, such as endolymphatic sac surgery, chemical labyrinthectomy (intratympanic gentamicin (ITG) injection), labyrinthectomy, and vestibular neurectomy, based on the patients’ residual hearing level. Chemical labyrinthectomy is a procedure that involves the ablation of vestibular type I hair cells using aminoglycosides. Since the introduction of chemical labyrinthectomy using streptomycin by Fowler in 1948, the procedures and drugs for chemical labyrinthectomy have been consecutively investigated [4]. Currently, gentamicin via intratympanic injection or direct middle ear application is the most frequently used treatment because previous studies have shown better treatment outcomes with minimal morbidity [5]. Although this procedure is convenient and has minimal morbidity, the risk of hearing loss from chemical labyrinthectomy using gentamicin remains a major concern for clinicians because gentamicin is toxic not only to vestibular hair cells but also to cochlear hair cells [6]. In humans, half of all patients who received repeated systemic high-dose aminoglycoside therapy showed high-frequency hearing loss [7]. The hearing level after chemical labyrinthectomy using gentamicin can be aggravated up to 31.08% depending on the administration and titration method [8,9]. Further, the short-term repeated gentamicin application showed a cumulative effect increasing the chance of hearing loss [10,11].

The drug administered to the middle ear passes through the round and oval window membranes into the inner ear. The round window is closer to the cochlea, but the oval window is close to both the cochlea and the vestibule. We hypothesized that concomitant application of dexamethasone and gentamicin on the round window and the oval window, respectively, can reduce hearing loss by acting mainly through the cochlea due to its proximity to the compartment. This hypothesis was based on research that reported that dexamethasone prevented gentamicin-induced hearing loss in animal models [12,13]. Furthermore, there can be a synergistic effect of gentamicin and dexamethasone in controlling vertigo attacks because the two drugs have different mechanisms of controlling vertigo [14]. Thus, the purpose of this randomized clinical trial is to investigate the effectiveness of concomitant intratympanic application of dexamethasone and gentamicin following middle ear exploration.

## 2. Materials and Methods

### 2.1. Patient Enrollment

This was a single-institutional, prospective, single-blinded, randomized clinical trial conducted between 11 November 2015 and 16 October 2021. This study was approved by the institutional review board of Severance hospital (Seoul, Korea) before patient enrollment (Project number 4-2016-0096). All participants provided written informed consent. Patients were eligible if they were: (1) 19 years or older; (2) diagnosed with definite MD according to the diagnostic criteria suggested by the Barany Society (2015); and (3) scheduled to undergo chemical labyrinthectomy for intractable vertigo attacks [1]. Patients with intractable vertigo were prescribed betahistidine and diuretics, along with lifestyle modifications for more than three months; additional treatment with intratympanic dexamethasone injection did not reduce the number of vertigo spells during following three months. A vertigo attack was defined as more than 20 min of spontaneous true-vertigo-type dizziness regardless of ear symptoms. Participants were excluded if they had a history of: (1) central vertigo; (2) head trauma; (3) other otologic disease (such as tympanic membrane perforation, otitis media, benign paroxysmal positional vertigo, and unilateral vestibulopathy of the other ear); and (4) suspected bilateral MD. One or two weeks prior to chemical labyrinthectomy, all participants underwent pure-tone audiometry and bithermal caloric test.

### 2.2. Patient Grouping, Randomization, and Power Analysis

The patients were randomized into two groups: gentamicin with normal saline (GO group) and gentamicin with dexamethasone (GD group). Randomization was performed using an online random number generator, with stratified block randomization performed by the research coordinator. The treatment allocation was concealed from the patient to mitigate bias. To calculate the sample size, G * Power software (Dusseldorf, Germany) was used. The expected mean difference of hearing threshold between the two groups was set at 10 dB and the standard deviation (SD) at 8 dB because a difference of less than 10 dB is possible in intra-subject variability of pure-tone audiometry [15]. The power analysis indicated that a sample size of at least 11 per group was required for an alpha level of 0.05 and a statistical power of 80%. Assuming a 20% failure rate, the target sample size was 13 in each group.

### 2.3. Surgical Procedure and Follow-Up

After administering local anesthesia to the external auditory canal, a tympanomeatal flap was elevated. Middle ear exploration was performed to identify the round and oval windows under a surgical microscope. If the surgical view was poor, a surgical endoscope was used. Pieces of gelatin sponge (Gelfoam^®^, Pfizer, Brooklyn, NY, USA) were soaked in gentamicin, dexamethasone, or normal saline. In the GO group, gentamicin-soaked Gelfoam^®^ pieces were applied to the oval window, and saline-soaked Gelfoam^®^ pieces were applied to the round window. In the GD group, gentamicin-soaked Gelfoam^®^ pieces were applied to the oval window, and dexamethasone-soaked Gelfoam^®^ pieces were applied to the round window.

The patients were scheduled to visit the outpatient clinic at 2, 4, and 8 weeks after the procedure. If the patient’s tympanic membrane was well healed at four weeks after the procedure, a pure-tone audiogram was performed at the next visit (eight weeks after the procedure). Thereafter, the patients regularly visited the clinic every 2–3 months and were asked whether they had any vertigo attacks until 12 months after the procedure. The vertigo attacks were defined as aforementioned.

### 2.4. Outcome Measurement

The parameter for primary outcome was the difference in the hearing preservation between the two groups. PTA_4_ (average threshold shift of 0.5, 1, 2, and 3 kHz frequencies) and PTA_high_ (average threshold shift of 4 and 8 kHz frequencies) were evaluated before and after the procedure. The PTA_high_ was separately evaluated because gentamicin ototoxicity may primarily affect the high-frequency region of the cochlea. In addition, the effect of gentamicin ototoxicity should be separated from the hearing fluctuation of MD, which is mainly in the low- to mid-frequencies. The secondary outcomes were (1) changes in the results of bithermal caloric test, (2) changes in the frequency of vertigo attacks (2–6 and 6–12 months after the procedure), and (3) the need for secondary treatment due to failure to control vertigo attacks.

### 2.5. Statistical Analysis

All continuous data were described as mean (SD). If the data passed the normality test (Shapiro–Wilk test), the Student’s t-test was used to compare the two groups; if not, the Mann–Whitney U test was used. The Wilcoxon matched-pairs signed rank test was used to compare the groups before and after the procedure. Fisher’s exact test (two-tailed) based on a contingency table was used to compare the proportion between the two groups. All statistical analyses were performed using SPSS version 25.0 (IBM, Armonk, NY, USA). The significance level was set at *p* < 0.05 for all statistical analyses.

## 3. Results

### 3.1. Information of Participants

**Figure 1:**
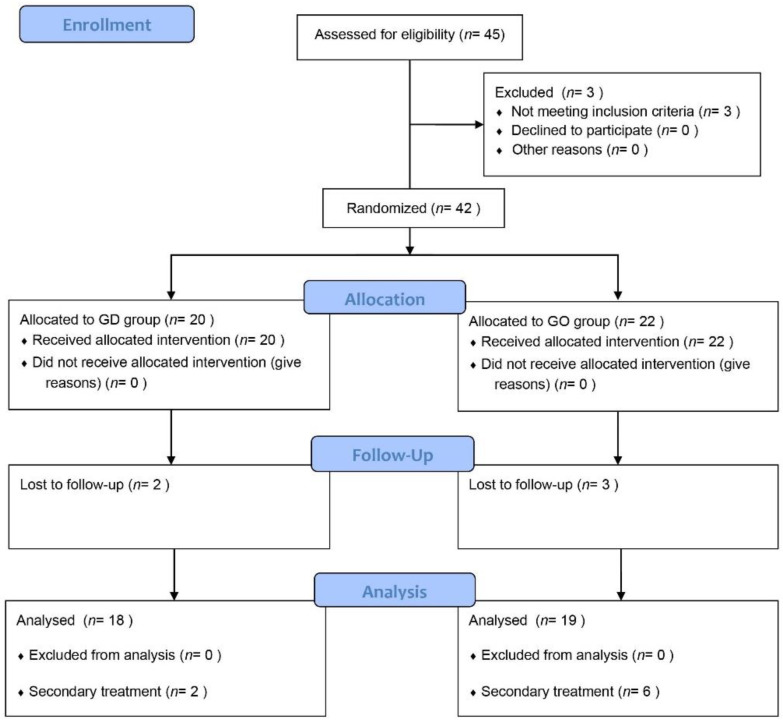
Flow diagram of study participants.

The enrollment of the participants began on 11 November 2015 and ended on 15 July 2020. A total of 37 participants were analyzed: 19 in the GO group and 18 in the GD group (Figure 1). Five participants (three in the GO group and two in the GD group) were lost during follow-up. According to inclusion/exclusion criteria, patients who are suspicious of vestibular migraine, autoimmune hearing loss, and familial history of MD were not included. None of the enrolled patients had surgical complications, including tympanic membrane perforation, post-operative infection, and taste change, at eight weeks after the surgical procedure. There was no statistically significant difference in age, sex, hearing level at each frequency, canal paresis value, or frequency of vertigo attacks between the two groups (Table 1). As expected, both groups showed moderate-to-severe hearing loss (51.38 dB and 49.17 dB in the GO and GD groups, respectively) and canal paresis (44.15% and 39.57% in the GO and GD groups, respectively).

### 3.2. Hearing Preservation

**Figure 2:**
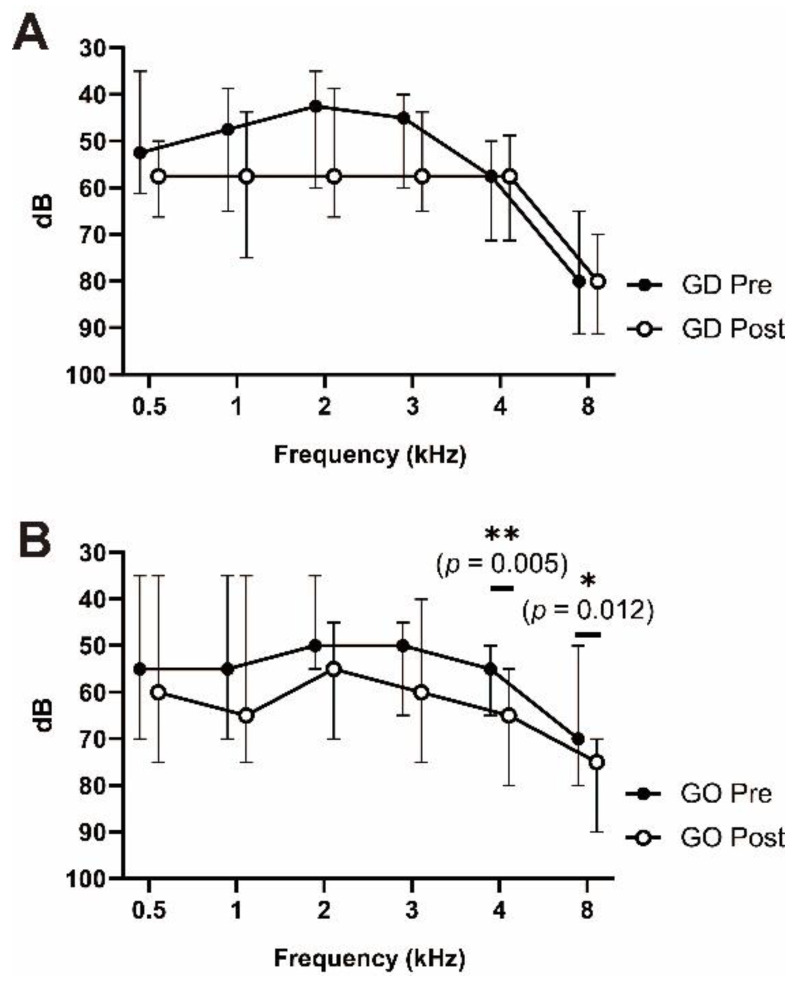
Hearing functions before and after chemical labyrinthectomy: (**A**) pure-tone audiogram of the GD group. (**B**) Pure-tone audiogram of the GO group. The high-frequency hearing thresholds (4 and 8 kHz) significantly worsened eight weeks after chemical labyrinthectomy only in the GO group. The black/hollow circles indicate median values. The error bars indicate the interquartile range. Wilcoxon matched-pairs signed rank test was used for analysis. GD: gentamicin combined with dexamethasone; GO: gentamicin combined with normal saline; Pre: before surgery; Post: 8 weeks after surgery. * *p* < 0.05, ** *p* < 0.01.

**Figure 3:**
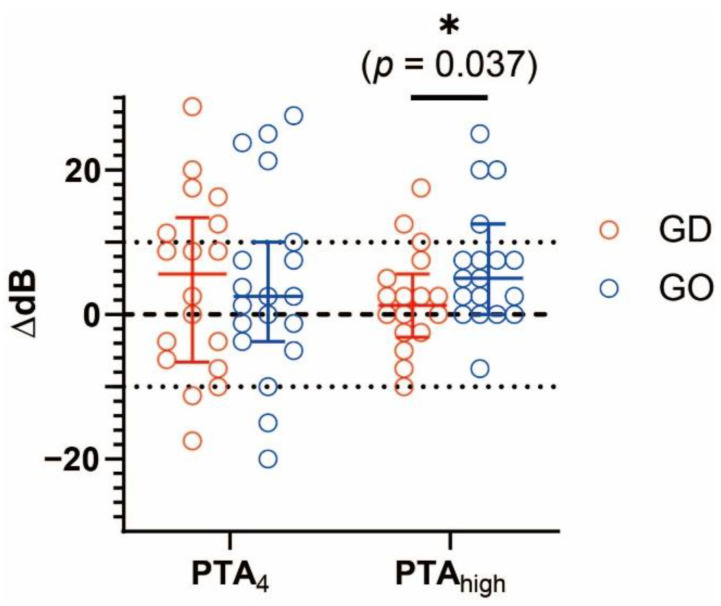
Change in hearing threshold in low- to mid-frequencies (PTA_4_) and high frequencies (PTA_high_). The average threshold shift after surgery was significantly larger in the GO group compared to the GD group. The circles indicate individual results of threshold shift. The large bar indicates the median value, and the error bars indicate the interquartile range. Mann–Whitney U test was used for analysis. Two individual results in PTA_high_ were outside the y-axis limits and not shown in the graph. GD: gentamicin combined with dexamethasone; GO: gentamicin combined with normal saline; PTA_4_: average threshold shift of 0.5, 1, 2, and 3 kHz frequencies; PTA_high_: average threshold shift of 4 and 8 kHz frequencies. * *p* < 0.05.

The paired analysis of hearing threshold before and after the procedure revealed that the procedure induced significant hearing loss at 4 and 8 kHz (*p* = 0.005 and 0.012, respectively) only in the GO group (Figure 2). There were no significant changes in the thresholds of all frequencies in the GD group. The change in thresholds after the procedure was significantly higher in PTA_high_ in the GO group (*p* = 0.037) but not in PTA_4_ (Figure 3). In particular, the threshold change was significantly higher at the 4 kHz frequency in the GO group (*p* = 0.049) (Table 2).

### 3.3. Secondary Outcomes

The average canal paresis in the bithermal caloric test was increased in both groups. The GD group showed a tendency to a larger increase in canal paresis (change by 6.24% vs. 21.05%), but the difference was not significant. Secondary treatment, including ITC and revision chemical labyrinthectomy, was required to treat vertigo in eight participants until the last follow-up (Table 3): two were in the GD group and six were in the GO group. The proportion of patients who needed secondary treatment was higher in the GO group, but the difference between the two groups was not statistically significant. We defined “treatment success” as the frequency of vertigo attacks being less than 40% compared to before chemical labyrinthectomy without secondary treatment (similar to classes A and B in the classification suggested by the 1995 American Academy of Otolaryngology–Head and Neck Surgery (AAO-HNS) guidelines) [16,17]. “Complete control” was defined as the absence of vertigo symptoms during the period (similar to class A in the classification suggested by the 1995 AAO-HNS guidelines). The GD group showed a significantly larger proportion of “treatment success” patients 2–6 months after the procedure (*p* = 0.019) and “Complete control” patients 2–12 months after the procedure (*p* = 0.038). However, the effects of both treatments diminished over time. Taken together, the GD group tended to show better dizziness control until 1 year after the treatment.

## 4. Discussion

In this prospective single-blinded randomized trial, chemical labyrinthectomy using gentamicin combined with dexamethasone showed a protective effect on high-frequency hearing level compared to the conventional method that uses gentamicin with normal saline. Furthermore, the combination method could more efficiently control the frequency of vertigo attacks until six months after the procedure. Given the results of this study, dexamethasone not only seems to have a protective effect on the ototoxicity of gentamicin, but also a synergistic effect with gentamicin to control vertigo.

Dexamethasone and gentamicin rapidly reach the perilymph fluid via passive diffusion through the round and oval windows after direct drug administration to the middle ear cavity [10,18,19]. Because both windows are in the basal turn of the cochlea, the drug concentration would have a gradient from basal to apical in which tonotopically high to low frequency of sound stimulation is detected. Thus, high-frequency hearing may be more sensitively affected by drug administration [11,20]. Gentamicin applied locally on the oval window, as in this study, diffuses to the vestibule more easily because it is close to the oval window. In contrast, the diffusion of dexamethasone (near the round window) to the vestibular organ is relatively difficult because there is no communication to the vestibular organ in scala tympani, but it easily diffuses to the cochlea due to the anatomical proximity. Therefore, local application of gentamicin and dexamethasone on the oval and round windows may be an ideal approach to control vertigo and preserve hearing. 

MD is well known to have characteristic fluctuating hearing loss in low- to mid-frequencies [1]. Our results also showed a larger SD of threshold shifts after the procedure at 0.5 and 1 kHz frequencies in both groups. This may be attributed to the fluctuating hearing of the enrolled patients with MD in low- to mid-frequencies. In addition, MD may have a large intra-subject variation in hearing and vestibular function based on the status of the disease phase. For instance, a patient in the ictal phase would have aggravated low- to mid-frequency hearing and canal paresis compared to a patient in the stable state. This can bias the analysis of PTA_4_ and canal paresis between the two groups. Thus, we additionally analyzed PTA_high_ to mitigate possible biases in the evaluation of the different effects of the two methods. This might also be useful in the evaluation of the hearing protection effect of dexamethasone against possible ototoxic effect of gentamicin because gentamicin is known to mainly affect high-frequency hearing levels [21].

The protective effect of dexamethasone on ototoxicity has been reported previously. In numerous animal studies, dexamethasone has shown a protective effect on ototoxicity induced by cisplatin and aminoglycoside [12,13,22]. Although the exact mechanism of gentamicin ototoxicity is unclear, the cell apoptosis pathway via reactive oxygen species (ROS) formation is thought to be mainly involved [23,24]. Dexamethasone is believed to decrease cell apoptosis via the nuclear factor kappa B pathway, as well as ROS formation [25,26]. This may be a critical mechanism that explains our results on high-frequency hearing preservation. Dexamethasone is presumed to reduce endolymphatic hydrops by increasing aquaporin; Na^+^ absorption from the endolymphatic space to the perilymphatic space by regulating epithelial Na^+^ channels; and Na, K-ATPase expression, which may contribute to vertigo control [27,28,29]. A previous systematic review reported that the ITC group had significant vertigo control compared to the placebo group [30]. Based on our result, the different mechanisms of dexamethasone and gentamicin may have a synergistic effect.

Previous studies on chemical labyrinthectomy using gentamicin to treat MD showed heterogeneous methods in the application of gentamicin, resulting in vertigo control and hearing preservation [9]. The administration methods of gentamicin are mainly categorized as intratympanic injection in aqueous form or use of sustained-release vehicles. Although sustained-release vehicles such as Gelfoam^®^ seem to provide precise and constant delivery of the drug, both methods were reported to show a similar effect on vertigo control (complete control rate of 70–80%) [9,31]. To date, studies comparing the treatment outcomes between intratympanic injection and sustained-release vehicles are sparse. The results of ITG and sustained-release vehicles should be analyzed with a consistent protocol in future studies. When comparing the results about the vertigo control to those of other studies described above, our results for the GO group are moderately satisfactory, with a success rate (less than 40% of the frequency of vertigo compared to the baseline during 2–12 months) of 52.6%. Although the exact reason for the relatively low vertigo control rate compared to other studies is not known, several factors can be considered. First, the application of gentamicin only in the oval window did not release a sufficient amount of gentamicin into the scala vestibule. Conventional intratympanic application of gentamicin can reach the vestibular organ via both the oval and round window, which may provide higher concentration compared to our method. The GD group might show a higher success rate for vertigo control due to the additional effect of dexamethasone to the effect of gentamicin. Second, dexamethasone diffused to scala tympani might impede the diffusion of gentamicin from scala vestibuli to scala tympani by increasing osmolality of scala tympani, which consequently causes higher gentamicin concentration in the scala vestibule. This might result in more vestibular ablation in the GD group than in the GO group. Although the change in canal paresis value was not statistically significant, the GD group showed relatively larger mean canal paresis value change than that in the GO group. Third, the number of patients enrolled in this study was not sufficient. For instance, canal paresis value changes showed relatively large standard deviation. Although several meaningful results were obtained from the study population, the study should be extended to a larger population to obtain more convincing results. However, the additional effect of concomitant application of dexamethasone with gentamicin on vertigo control and hearing protection seems promising; therefore, dexamethasone can be used with other gentamicin administration methods. In the future, the outcomes of serial administration of ITC following ITG could be investigated in further studies.

The limitation of this study was the short follow-up period, which was up to 12 months after the procedure. Therefore, vestibular results could not be concluded in this study. Considering that MD spontaneously regresses over time, the treatment outcome of this study might be underestimated when compared to studies that have reported a longer follow-up period [32]. Especially for ITC, some studies reported the short-term effect six months after the injection; our result also reflected this short-term advantage of ITC [33]. We thought that short-term effect evaluation of intratympanic dexamethasone in vertigo control and hearing preservation is more appropriate because the concentration of dexamethasone has a half-life of several hours of in the cochlea, as reported in an animal experiment [34]. Although the effect of dexamethasone is expected to last only a short time, a long-term follow-up result of more than two years should also be investigated in future studies.

In conclusion, the direct middle ear administration of gentamicin combined with dexamethasone protects high-frequency hearing and shows better short-term vertigo control compared to gentamicin combined with normal saline.

## Figures and Tables

**Table 1:** Baseline characteristics of study participants.

Characteristic	GO Group (*n* = 19)	GD Group (*n* = 18)	* *p*-Value
Age, mean (SD), years	55.05 (16.06)	54.78 (15.13)	0.958 ^a^
Sex, male (%)	7 (36.8)	8 (44.4)	0.743
PTA_4_, mean (SD), dB	51.38 (18.39)	49.17 (16.56)	0.703 ^a^
0.5 kHz	53.42 (21.48)	51.67 (17.90)	0.789 ^a^
1 kHz	53.16 (20.63)	50.83 (18.81)	0.723 ^a^
2 kHz	47.89 (18.66)	45.83 (16.56)	0.725 ^a^
3 kHz	51.05 (17.92)	48.33 (17.41)	0.643 ^a^
PTA_high_	59.34 (21.55)	67.36 (19.26)	0.223 ^b^
4 kHz	52.89 (20.02)	56.94 (18.56)	0.580 ^b^
8 kHz	65.79 (24.28)	77.78 (22.18)	0.126 ^a^
CP value in caloric test before surgery, mean (SD), %	44.15 (27.19)	39.57 (24.22)	0.593 ^a^
Frequency of vertigo attacks, mean (SD), per month	1.70 (1.27)	1.97 (1.46)	0.512 ^b^
Disease duration to surgery (SD), days	1723 (1524)	1908 (1619)	0.663 ^b^

^a^ Student’s t-test, ^b^ Mann–Whitney U test, * *p* < 0.05. GO: gentamicin combined with normal saline; GD: gentamicin combined with dexamethasone; SD: standard deviation; PTA_4_: average threshold shift of 0.5, 1, 2, and 3 kHz frequencies; PTA_high_: average threshold shift of 4 and 8 kHz frequencies; Frequency of vertigo attacks: average number of vertigo attacks per month during recent 6 months before chemical labyrinthectomy.

**Table 2:** Changes in the results of pure-tone audiometry and caloric test after chemical labyrinthectomy.

Characteristic	GO Group (*n* = 19)	GD Group (*n* = 18)	* *p*-Value
PTA_4_, mean (SD), ΔdB	3.95 (13.22)	4.17 (12.51)	0.959 ^a^
0.5 kHz	2.63 (18.44)	3.61 (15.79)	0.864 ^a^
1 kHz	3.68 (16.15)	4.72 (15.38)	0.843 ^a^
2 kHz	4.21 (11.82)	5.28 (12.77)	0.817 ^b^
3 kHz	5.26 (13.49)	3.06 (12.85)	0.963 ^b^
PTA_high_, mean (SD), ΔdB	8.82 (12.81)	−0.42 (12.52)	0.037 ^b^*
4 kHz	8.42 (14.25)	−1.39 (13.91)	0.049 ^b^*
8 kHz	9.21 (15.12)	0.56 (13.49)	0.174 ^b^
Change of CP value in caloric test, mean (SD), Δ%	6.24 (31.95)	21.05 (23.30)	0.118 ^a^

ΔdB: difference in the hearing threshold between the values before and after chemical labyrinthectomy; Δ%: difference in the canal paresis value in caloric test before and after chemical labyrinthectomy; CP: canal paresis; ^a^ Student’s *t*-test; ^b^ Mann–Whitney U test. * *p* < 0.05.

**Table 3:** Vertigo control results after chemical labyrinthectomy.

Characteristic	GO Group (*n* = 19)	GD Group (*n* = 18)	* *p*-Value
Secondary treatment required (%)			
2–6 months	4 (21.05)	1 (5.56)	0.340
2–12 months	6 (31.58)	2 (11.11)	0.232
Complete control (%) ^a^			
2–6 months	8 (42.11)	11 (61.11)	0.330
2–12 months	3 (15.79)	9 (50.00)	0.038 *
Treatment success (%) ^b^			
2–6 months	11 (57.89)	17 (94.44)	0.019 *
2–12 months	10 (52.63)	15 (83.33)	0.079

^a^ Absence of vertigo during the period. ^b^ Less than 40% of the frequency of vertigo compared to baseline during the period. * *p* < 0.05.

## Data Availability

Data sharing not applicable.
